# ‘It is easier to not allow them to see your disability straight away, to see you as a person’: An Interpretative Phenomenological Analysis of video gaming from the perspectives of men with Duchenne Muscular Dystrophy

**DOI:** 10.1177/02692163231172246

**Published:** 2023-05-02

**Authors:** George Peat, Alison Rodriguez, Joanna Smith

**Affiliations:** 1 School of Healthcare, University of Leeds, Leeds, UK; 2 Health Sciences Department, Martin House Research Centre, University of York, York, UK

**Keywords:** Video games, Duchenne Muscular Dystrophy, qualitative, Interpretative Phenomenological Analysis, wellbeing, hospice care

## Abstract

**Background:**

Young men with Duchenne Muscular Dystrophy benefit from palliative care that supports their psychosocial needs. Acknowledging the sub-cultures they engage with can support their wellbeing. Anecdotal reports suggest video gaming is a sub-culture engaged with by young men with Duchenne Muscular Dystrophy.

**Aim:**

To explore the lived experience of video gaming from the perspective of young men with Duchenne Muscular Dystrophy.

**Design:**

Interpretative Phenomenological Analysis approach involving in-depth interviews using a topic guide that focused on social media broadly, with reference to video gaming. Sequential interviewing was undertaken to support participation regarding fatigue and tiredness, symptoms of Duchenne Muscular Dystrophy.

**Setting/participants:**

Participants were purposefully recruited from a hospice in the North of England. Twitter was used to support recruitment. Eight young men with Duchenne Muscular Dystrophy were recruited to the study.

**Results:**

Five themes were developed; ‘gamer as a shared and accepted identity’, ‘an existential and bodily escapism’, ‘introspection through video gaming’, ‘video gaming as a release’ and ‘when life gives you few choices-video game’. Motivations for engagement with video gaming are diverse and reflective of the situated perspectives of young men with Duchenne Muscular Dystrophy.

**Conclusions:**

An awareness of the popular sub-cultures that young men with Duchenne Muscular Dystrophy engage with is key to building a therapeutic alliance, establishing rapport and recognising personhood in interactions between professionals and persons in palliative care settings. This study highlights the value of video gaming, offering professionals valuable insight into its placement in the daily lives of young men with Duchenne Muscular Dystrophy.

**What is already known on the topic**

- Anecdotal reports suggest video gaming is an important activity in the daily lives of young men with Duchenne Muscular Dystrophy.
- Previous literature reports findings related to accessibility challenges and their impact on video game experience, gamer demographics and motivations for video game engagement.
- Video gaming facilities are increasing in clinical and hospice settings thanks to charity organisations.

**What this paper adds?**

- Video gaming supports young men with Duchenne Muscular Dystrophy to engage with other peers based on a shared and accepted identity.
- Young men with Duchenne Muscular Dystrophy are able to escape their everyday lives through video gaming and engage in activity otherwise unavailable to them.
- Young men with Duchenne Muscular Dystrophy face barriers to engaging in social activity outside of video gaming.

**Implications for practice, theory or policy**

- Video gaming has value both in and outside of clinical settings to support the wellbeing of young people with Duchenne Muscular Dystrophy and disabled young people more broadly.
- While we advocate for video gaming as a meaningful and enjoyable activity, it is not a solution to the challenges young men with Duchenne Muscular Dystrophy can face to living fulfilling young adult lives.
- An awareness of the popular sub-cultures such as video gaming that young men with Duchenne Muscular Dystrophy engage with can support rapport building and recognition of personhood in interactions with professionals and persons in palliative care settings.

## Background

Since its inception, video gaming has become a popular activity shared by children and adults worldwide. Much has been written about player motivations for gaming, however relatively little attention has been given to the experiences of disabled gamers, despite anecdotal reports suggesting a significant proportion regularly engage with video gaming.^1–4^ In this paper, we draw on the findings of an Interpretative Phenomenological Analysis (IPA) study with eight young men with Duchenne Muscular Dystrophy to understand their experiences of video gaming.

Duchenne Muscular Dystrophy is a life-limiting neuromuscular condition with an estimated prevalence of 15.9–19.5 per 100,000 births.^5,6^ The condition almost exclusively effects males and is characterised by progressive muscle degeneration and weakness due to a defect in dystrophin, a protein necessary for muscle cells to function correctly.^5^ Typically, by the age of 12, children diagnosed with Duchenne Muscular Dystrophy are non-ambulant.^7^ Relatively recent advances in respiratory, cardiology and gene therapy have significantly increased their life expectancy, with people with Duchenne Muscular Dystrophy now likely to live into their 30s and 40s, and potentially beyond.^5,8,9^ However, living with Duchenne Muscular Dystrophy is challenging, and requires multidisciplinary health and social care input to support young men living with the condition to have meaningful and fulfilling lives.

Video and computer gaming are congruous with forms of social media, including social networking sites and interactive blogging, in their capacity to bring people together through their online functionality and player connectivity. Research into video gaming, and social media use more broadly by young men with Duchenne Muscular Dystrophy is limited. In 2018, we published the findings of an integrative review of research that focused on social media use by adolescents and young adults with life-limiting and life-threatening conditions. Of the 15 studies identified, 14 reported findings on social media use by adolescents and young adults with cancer. End-stage renal disease was addressed by the remaining article. No other illnesses representative of life-limiting or life-threatening conditions were found, despite a comprehensive search of multiple databases. No studies on social media use by young people with neuromuscular conditions such as Duchenne Muscular Dystrophy were identified.^10^

An expanded search of the literature identified an additional three studies on video gaming use by young people with physical disabilities.^11–13^ Of these studies, two were survey-based,^11,13^ and one interview-based.^12^ Findings primarily related to accessibility challenges and their impact on video-gaming experiences, understanding the demographics of disabled gamers and understanding motivations for video game engagement. A separate body of literature has explored the potential utility of gaming to support physical assessment and physiotherapy of Muscular Dystrophy and related conditions.^14–16^ However, no studies within this evidence base have focused specifically on the experiences of video gaming from the perspectives of young men with Duchenne Muscular Dystrophy.

Despite a paucity in empirical evidence, anecdotal and social media reports suggest video/computer gaming is a popular and meaningful activity undertaken by young men with Duchenne Muscular Dystrophy.^1–4^ For example a young man with Duchenne Muscular Dystrophy, who was an active blogger, poignantly summarised the meaning of video gaming as: ‘*my chains are broken and I can be whoever I want to be. In there I feel normal*’.^4^ To support the wellbeing of men with Duchenne Muscular Dystrophy, it is necessary to understand their experiences of engaging in daily activity such as video gaming. To address the dearth in evidence into video gaming use by this patient group, this study aimed to explore the lived experience of video gaming from the perspectives of young men with Duchenne Muscular Dystrophy.

## Methods

Interpretative Phenomenological Analysis (IPA) underpinned the study and informed the methods and rationale for recruitment, data collection and analysis. IPA was appropriate because it is fundamentally concerned with how a particular phenomenon (video gaming) comes to mean something to a particular person/group (young man/young men with Duchenne Muscular Dystrophy), in a particular situated context (the everyday life living with Duchenne Muscular Dystrophy).^17^ Ethical approval was granted by the University of Leeds School of Healthcare Research Ethics Committee (Reference: HREC17-009).

### Setting

In the United Kingdom (UK), young men with Duchenne Muscular Dystrophy and their families are typically offered hospice care, which provides a range of support including psychosocial support and respite care. A hospice in the North of England supported the study and recruitment. The social media platform Twitter was also used to support recruitment.

### Population

Young men with Duchenne Muscular Dystrophy were included if they met the criteria defined in Table 1.

**Table 1.** Inclusion and exclusion criteria.

Inclusion criteria	Exclusion criteria
18–35 years old	Aged less than 18 years
Diagnosed with Duchenne Muscular Dystrophy	Those who lack the mental capacity to participate in the study, guided by the 2005 Mental Capacity Act
Interacted or engaged with video gaming	

### Sampling

Young men aged 18–35 with Duchenne Muscular Dystrophy with an interest in video gaming were eligible for the study. Purposive sampling was undertaken by professionals at a children and young persons hospice, as per the study inclusion criteria. Relatedly, purposive sampling was undertaken by GP to identify eligible participants who responded to the Twitter advertisement.

### Recruitment

The lead nurse sent recruitment packs to potential participants 1 month prior to a planned respite stay at the hospice, that included an invite letter, information sheet, and a copy of the consent form. Those interested in participating were asked to contact the lead researcher (GP) using details provided in the information sheet. Email dialogue between potential participants and GP was established prior to the interview, with a date/time for interview subsequently agreed. To support recruitment, @Twitter (social media platform) was utilised with tweets (messages) posted to advertise the study, developed in consultation with patient and public involvement (PPI) representatives. Profiles of relevant charities and organisations were added to the tweets to increase tweet visibility. The tweets received a total of 88 engagements, including 18 retweets and 12 likes. Contact details of GP were provided in the tweet and people who expressed an interest in taking part in the study contacted GP and were then sent a recruitment pack by email.

### Data collection

Data were collected by semi-structured in-depth interviews to explore participants’ experiences of social media broadly, with video gaming a featured topic. Fatigue and tiredness are common symptoms of Duchenne Muscular Dystrophy.^17^ To support participation in the study, interviews were offered to be undertaken sequentially at more than one time point. An interview guide was developed from the literature, theory, the findings of a Twitter Chat and in consultation with two PPI representatives and a young person’s advisory group.^18^ Topics included the placement of video gaming in the daily lives of participants, the value and utility of video gaming and the barriers or challenges to engagement with video gaming. Most interviews took place face-to-face at the participant’s home, or at the hospice where they were receiving respite care, with one participant interviewed online (via Skype following university protocols). Video gaming was used in several of the initial interviews to develop rapport with participants and to support the elicitation of video gaming experiences. All interviews were undertaken by GP. With participant’s consent, interviews were audio-recorded and transcribed verbatim. Interviews took place between October 2018 and July 2019.

### Data analysis

Interpretative Phenomenological Analysis was undertaken.^19^ An initial case-by-case approach was adopted, whereby each participant account was analysed individually. To aid data familiarisation, interview recordings were listened to and transcripts read multiple times, with initial thoughts noted. A process of developing descriptive, linguistic and conceptual codes followed. Descriptive codes highlighted content that appeared to ‘matter’ to participants. Engaging in descriptive coding helped to structure participant narratives. Linguistic coding identified how participant’s utilised pronouns, laughter, pauses and repetitive discourse to convey their narrative. Finally, conceptual coding involved an interpretive probing and questioning of the data. Descriptive, linguistic and conceptual codes were then grouped into themes that summarised participant accounts. The stages of familiarisation, coding and theme development were repeated for each participant account. Prior to moving to the next account, interpretations (thoughts, assumptions and ‘fore-meanings’)^20^ developed through engaging with the previous account were documented to support the analysis of each account.^19^ On completion of the case-by-case analysis, an iterative process of identifying patterns and divergence across cases followed, resulting in new cross-case themes. Cross-case themes were then grouped to develop superordinate themes that structured the overall findings. Superordinate theme development was debated and subject to on-going refinement through regular meetings between GP, AR and JS.

### Researcher characteristics and reflexivity

Prior to developing the study, GP had no experience of engaging with young men with Duchenne Muscular Dystrophy or similar life-limiting/threatening conditions. During study development, GP spent a period of time at a hospice, to understand the needs and priorities of young men with Duchenne Muscular Dystrophy. GP has a background in psychology, with experience in qualitative methodology and methods. The study was undertaken as part of a PhD qualification. GP enjoys video gaming as a hobby, and was therefore able to share experiences with participants, supporting rapport. GP regularly journaled throughout the study, questioning and bringing to fore his assumptions/presuppositions about the sample and topic area.

The study was supported by two PPI representatives who held senior nursing roles at a children and young adult hospice/s and were able to appropriately guide and inform the study. AR and JS both have extensive experience of delivering research in the field of young person’s palliative care/long-term conditions and could offer GP relevant support.

## Results

### Sample

A sample of eight young men with Duchenne Muscular Dystrophy were interviewed. Young men living with Duchenne Muscular Dystrophy experience greater fatigue in comparison to typically developing males.^17^ Therefore, to support their engagement, the sample were interviewed at more than one time point. This also engendered rapport with participants that supported in-depth discussions about their experiences. In total, 18 interviews were undertaken with each interview lasting on average 40 min. The characteristics of the sample are outlined in Table 2.

**Table 2.** Participant characteristics.

	*n*	%
*Sex*		
Male	8	100
Female	0	
*Age*		
18–21	3	40
22–25	3	40
25+	2	20
*Ethnicity*		
White British	5	62.5
British Asian	3	37.5
*Neuromuscular condition*		
Duchenne Muscular Dystrophy	8	100

### Thematic description of findings

The meaning of video gaming is illustrated by five themes; ‘gamer as a shared and accepted identity’, ‘an existential and bodily escapism’, ‘introspection through video gaming’, ‘video gaming as a release’ and ‘when life gives you few choices-video game’.

### Theme 1: ‘Gamer’ as a shared and accepted identity

Video gaming provided a sense of identity that could be shared with others without prejudice. Participants described themselves as ‘gamers’ and opted to prioritise their ‘gaming self’ when describing themselves to others:

. . .would probably put gamer and the types of games I play, that would be it really. Lee.

Video gaming offered an alternate landscape for self-presentation. Participants described the social benefit of being able to ‘hide’ their disability from other gamers, and form friendships with others based on *‘other things than just your disability’ (Vivek).* Avatars and gaming characters appeared as modalities that offered choice and freedom over aspects of self revealed to others:

Well I think it is easier to kind of not allow them to see your disability straight away. To see you as a person and a gamer. I realised that you formed friendships that are about a lot of other things than just your disability. Vivek.

Participants described challenges in presenting a self in line with how they wished to be viewed by others outside of video gaming. Periods of bullying and teasing targetted at their disability were associated with difficult times where they were considered as Beings with *‘summet wrong with them’* (Tom).

In summary, gaming facilitated additional identity formation without an association to their disability, allowing them to feel fully participant in the virtual world and not judged by those with ableist ideologies.

### Theme 2: An existential and bodily escapism

Engagement with video gaming offered participants existential and bodily experiences of escapism. Video gaming provided a space where they could *‘forget’*, *‘shut-out’* or *‘escape’* from life outside of the video game. In this sense, escapism was experienced existentially, with gaming providing a pause from life as lived outside of gaming:

. . .gaming kind of helped me to take my attention away from negative things all the time about my weakness, because it felt like I was grieving each time I had a little bit of deterioration but having the game there to play I forgot about that (deterioration). Vivek.

On an interrelated level, participants video gamed to experience activity *‘that I (they) wouldn’t be able to do in real life’ (Simon).* Participants described a range of activities including driving, horse riding and sports such as football and mixed martial arts that were perceived as outside their reach. Other activities outlined included more intimate experiences such as washing and grooming:

I like the driving ones obviously I don’t drive so it’s like a thing I get to know exactly what it is like to drive, well ye know not quite exactly but. . . Simon

. . .but when you fall you get muddy, yeah and then you can wash yourself, whereas I wouldn’t be able to do that in real life, so it is just experiencing new things. Vivek

Escapism in these instances therefore appeared to mean a departure from the body as experienced outside of video gaming, to a transformed bodily existence, akin to an *‘astral projection’* (Vivek).

In summary, participants found meaning in the ability of video games to offer them a haven and space to escape to during periods of difficulty. Likewise, the activity offered a medium to experience activity otherwise perceived as out of reach in their offline worlds.

### Theme 3: Introspection through video gaming

Gaming provided a space for participants to make sense of and ponder their sense of self and identity. Participants described looking inward to question, ponder and evaluate their self. Exposure to activities through video gaming otherwise unavailable provided participants with a different world view. Participants reflected on how life would appear differently if activities accessible through gaming were present offline, for example the ability to walk. Relatedly, participants compared their lives to those of gaming characters. Some participants perceived that the lives of gaming characters were *‘a bit more exciting than mine’* and therefore wished *‘to be some of the characters that I am in games’* (Simon).

Yeah would I drive like I do on GTA (video game) and do the stuff I am doing in the game. Ye know what I mean. . .cos in my case I have never been able to walk. Ye know what I mean I have never had that. So there is always that thing of what would I be like if I could walk? Mohsin.

I think with everything I do like gaming. . .I kind of start to understand myself more, it is kind of a way to like create an identity and kind of shape it. So yeah I think that is what gaming is, it is shaping you as a person. Vivek.

In summary, video gaming offered participants’ experiences otherwise unavailable outside of gaming. Access to these experiences promoted introspection that in turn supported them to make sense of their identities and lives.

### Theme 4: Video gaming as a release

Video gaming offered participants a space to release emotions that otherwise would negatively impact their wellbeing. Participants used discourse such as *‘vent out’, ‘taking out’* and *‘get rid of’* to describe the cathartic quality of video gaming:

. . .and I used it (video gaming) as a coping mechanism as I got older. Mainly because as a kid I was confused, I had a lot of anger, ye know about myself and the position I am in. I just didn’t understand why, so I used Tekken (game) to vent out my frustrations. Mohsin.

Participants identified video gaming as a viable space to make sense of emotions associated with their daily lives. Participants perceived that discussing daily stressors and challenges to significant others such as their parents may have upset them, opting instead to process these emotions through video gaming:

Ye know cos they (parents) might think I don’t know they’ve failed you or something like that. When they haven’t but. . .. Tom.

In summary, video gaming appeared to be a crucial activity to support wellbeing, offering a space to make sense of emotions that manifested in participants’ everyday lives, and *‘vent out’* challenging or difficult emotions.

### Theme 5: When life gives you few choices-video game

The presence of video gaming in participants’ lives was associated with a dearth in opportunities outside of the activity. Participants described being *‘. . .isolated from the real world’* (Mohsin), something they attributed to few avenues to socialise and family dynamics they described as over-protective:

I mean you don’t understand if you’ve got a disability, and you’ve got an overprotective family, that don’t let you play out because they are scared that you are going to get hurt, or that you are going to get picked on, or ye not going to fit in, the only thing you can do is gaming. Mohsin

Yeah because people who can go to the gym or do sport and things you get to know people through that stuff ye know going out, but I can’t really go out on nights out and stuff like that. Cos there is just no facilities or disabled access and stuff it all comes down to that. . . Tom

Video gaming held a consistent and daily presence in the lives of several participants *‘from whatever time I get up till I go to sleep’* (Lee). Participants’ were aware of their reliance on video gaming for everyday meaning, and voiced that if opportunity for other activities existed, their relational dynamic with video gaming would be significantly altered:

If I could I would not just game, if I could get out and do all that stuff I would hardly ever go on my Xbox. Tom.

In summary, the presence of video gaming in participants lives existed not always through choice, but through a belief based on experience that few other opportunities existed for meaningful activity and engagement.

## Discussion

### Main findings

Young men with Duchenne Muscular Dystrophy cite a range of motivations for daily engagement with video gaming. However, they were unanimous about its significance and importance in their everyday lives.

Our findings highlight how young men with Duchenne Muscular Dystrophy can tap into the shared and accepted identity of a gamer to portray a ‘narrative of non-difference’,^21^ to compete as equals and interact with peers. In addition, we found that video gaming can act as a space of solace and refuge for young men with Duchenne Muscular Dystrophy from the challenges of their everyday lives. The ability to transcend their everyday lives and immerse themselves within video games can aid wellbeing, offering the opportunity to process and make sense of troubling emotions.

Video gaming occupied a significant space in the lives of participants, with some stating it was their main daily activity. While citing the benefits of video gaming to their wellbeing, participants also recognised a balance between gaming life and life outside of gaming should exist. However, participants felt that few options exist other than to video game. Therefore, while our findings illustrate the benefits of video gaming to young men with Duchenne Muscular Dystrophy and those with life-limiting/threatening conditions more broadly, they do not underplay the importance of support and provision to enable young men with Duchenne Muscular Dystrophy to live fulfilling and meaningful young adult lives both in, and outside of video gaming.

### What this study adds

Previous study on video gaming use focused on accessibility barriers, gamer demographics and gamer motivation, with an underrepresentation of young men with Duchenne Muscular Dystrophy despite anecdotal reports suggesting the utility of the activity in their everyday lives.^10–14^ Adopting an in-depth phenomenological approach, this study adds new insight to the evidence base, highlighting the varied benefits of video gaming, demonstrating its placement and significance in the daily lives of young men with Duchenne Muscular Dystrophy.

Opportunities to socialise and connect with others can often be limited for young men with Duchenne Muscular Dystrophy.^21,22^ Leisure and social activities are often family-centred, with few opportunities to develop independent and active social lives.^21,22^ Furthermore, societal abliest ideologies have ‘othered’ and marginalised disabled young adults, confounding physical barriers such as poor accessibility to hinder social participation. Our findings concur and highlight experiences of bullying and derogatory behaviour experienced by young men with Duchenne Muscular Dystrophy. To negate physical and societal oppressions of social self, our findings show how young men with Duchenne Muscular Dystrophy tap into a popular gaming culture to engage with others based on the shared identity of a gamer. It is important that video gaming is recognised as an anchor into a popular shared identity that young men with Duchenne Muscular Dystrophy, and those with disabilities more widely, can utilise to support social interaction.

Studies have to date focused on the physical benefits of video gaming in areas such as physiotherapy.^14–16^ Whilst not underplaying the value of these studies, we found gaming has cathartic and therapeutic qualities that can support the emotional wellbeing of young men with Duchenne Muscular Dystrophy. Living as a young person with a degenerative condition can be challenging. At a time when other young men are becoming increasingly independent, those with Duchenne Muscular Dystrophy experience increasing dependence on both technology such as mobility chairs and ventilation equipment, and people to support their daily needs.^21–24^ We found video gaming offers a space for young men with Duchenne Muscular Dystrophy to make sense of their everyday experiences. Identifying the capacity of video gaming to support emotional wellbeing is significant, particularly because the population are at increased risk of anxiety and depression.^25^

The presence of video gaming equipment in clinical settings has increased in recent years largely due to the work of charity organisations both in the UK^26^ and internationally.^27^ To maximise the potential of this equipment, clinicians need to be aware of the therapeutic, cathartic and social benefits of video gaming, illustrated in our findings. Furthermore, we also found video gaming supported rapport between participant and researcher prior to and during interview/s, a finding also reported elsewhere.^28^ There is a growing emphasis on including young people with life-limiting/threatening conditions in discussions about their care.^29^ Clinicians may look to utilise video gaming as a tool to connect with patients and support involvement in discussions about their care. Further study is warranted to explore the potential utility of video gaming within clinical settings.

To support the wellbeing of young men with Duchenne Muscular Dystrophy, a balance should exist between video gaming as a source of enjoyment and occasional refuge, and ensuring it does not claim superiority over other realities. Extended periods devoted to video gaming can isolate young men with Duchenne Muscular Dystrophy from social groups and settings outside of video gaming realities.^5^ We found that participants recognised the need to balance gaming with other social activities. Nevertheless, participants consistently faced barriers to meaningful activity outside of video gaming, increasing their dependence on the activity. Our findings therefore complement a call for a ‘systemic cultural shift’ in the way young men with Duchenne Muscular Dystrophy and other children and adults with special educational needs and disabilities are viewed and supported.^30^

### Strengths and limitations

To the best of our knowledge, this is the first UK study to explore video gaming use by young men with Duchenne Muscular Dystrophy. This is significant because to support young men with Duchenne Muscular Dystrophy to live fulfilling and meaningful lives, it is necessary to better understand their daily lives and activities. A limitation of this study was the sample size recruited. However, the sample recruited was in keeping with an interpretive phenomenological approach.^17^ More so, the testimony of the study sample is supported by anecdotal reports of gaming use by this patient group,^4^ and previous study into gaming use by people with physical disabilities.^11–13^ Consequently, it is likely that the findings of the study are transferable to the wider population.

## Conclusion

Young men with Duchenne Muscular Dystrophy engage with video gaming to situate themselves as part of a popular sub-culture, to experience escapism, for a cathartic release, to make sense of their Being, and because few other opportunities for meaningful engagement exist. These findings can be implemented into practice, utilising gaming equipment available in hospital and hospice settings to connect, engage and provide escapism for young people in palliative settings. Relatedly, an awareness of the popular sub-cultures that young men with Duchenne Muscular Dystrophy engage with is key to building therapeutic alliance, establishing rapport and recognising personhood in interactions between professionals and persons in palliative care settings. More broadly, while we advocate for video gaming as a meaningful and important activity, it is not a solution to the challenges faced by this group to live fulfilling lives. Young men with Duchenne Muscular Dystrophy should not feel such an urge to escape or forget, nor should they require a space to release negative emotion, and nor should they compare themselves to software constructed characters and find themselves wanting. Future research and practice policy should be directed towards supporting the population to live fulfilling lives.

## Supplemental Material

sj-pdf-1-pmj-10.1177_02692163231172246 – Supplemental material for ‘It is easier to not allow them to see your disability straight away, to see you as a person’: An Interpretative Phenomenological Analysis of video gaming from the perspectives of men with Duchenne Muscular Dystrophy Click here for additional data file. Supplemental material, sj-pdf-1-pmj-10.1177_02692163231172246 for ‘It is easier to not allow them to see your disability straight away, to see you as a person’: An Interpretative Phenomenological Analysis of video gaming from the perspectives of men with Duchenne Muscular Dystrophy by George Peat, Alison Rodriguez and Joanna Smith in Palliative Medicine
