# Supplementary material for: ‘It is easier to not allow them to see your disability straight away, to see you as a person’: An Interpretative Phenomenological Analysis of video gaming from the perspectives of men with Duchenne Muscular Dystrophy
Source: Palliat Med. 2023 May 2;37(7):1016–24. doi: 10.1177/02692163231172246 (PMC10333970; doi:10.1177/02692163231172246)
Supplement: sj-pdf-1-pmj-10.1177_02692163231172246 – Supplemental material for ‘It is easier to not allow them to see your disability straight away, to see you as a person’: An Interpretative Phenomenological Analysis of video gaming from the perspectives of men with Duchenne Muscular Dystrophy [file sj-pdf-1-pmj-10.1177_02692163231172246.pdf]

Thematic table

Example extract one	Example extract two	Example extract three	Example descriptive, linguistic, and conceptual codes across cases	Theme
'would probably put gamer and the types of games I play, that would be it really' Lee	'Well I think it is easier to kind of not allow them to see your disability straight away. To see you as a person and a gamer'. Vivek	'Ye know what with a lot of people it helps their confidence, playing online, talking to likeminded people'. Mohsin	Talking to likeminded people<br>Gamer<br>To see you as a person<br>Not allow 'them' inference of the 'other<br>To be seen	'Gamer' as a shared and accepted identity
'…gaming kind of helped me to take my attention away from negative things all the time about my weakness, because it felt like I was grieving each time I had a little bit of deterioration but having the game there to play I forgot about that (deterioration)'. Vivek.	'I like the driving ones obviously I don't drive so it's like a thing I get to know exactly what it is like to drive, well ye know not quite exactly but…' Simon	'It's like I just find it amazing that you can go wherever you want'. Lee	'forget', 'shut out', 'escape'<br>Bodily transformation, to know what it is like'<br>'well ye know not quite exactly'<br>Limitless possibility<br>Not confined<br>To be taken away/distant from	An existential and bodily escapism
'Yeah would I drive like I do on GTA and do the stuff I am doing in the game. Ye know what I mean…cos in my case I have never been able to walk. Ye know what I mean I have never had that. So there is always that thing of what would I be like if I could walk?' Mohsin.	I think with everything I do like gaming…I kind of start to understand myself more, it is kind of a way to like create an identity and kind of shape it. So yeah I think that is what gaming is, it is shaping you as a person'. Vivek.	'I just think I would love to be some of the characters that I am in games and stuff. I just think ugh, probably because their life is a bit more exciting than mine ye know what I mean' Simon	Questioning of self<br>'What would I be like'<br>Comparative between life of avatar and self<br>Understanding of self<br>Shaping you as a person<br>Identity	Introspection through video gaming
"…and I used it (video gaming) as a coping	'Well I think like for Xbox it is a way of like sort of taking	'I suppose some games where you can get quite	Coping mechanism<br>Cathartic	Video gaming as a release

mechanism as I got older. Mainly because as a kid I was confused, I had a lot of anger, ye know about myself and the position I am in. I just didn't understand why, so I used Tekken (game) to vent out my frustrations". Mohsin.	out my stress and stuff'. Simon	involved, like angry as so it is quite a good way to like get rid of anger as well'. Tom	Stress release<br>Rid of anger<br>Venting out	
'If I could I would not just game, if I could get out and do all that stuff I would hardly ever go on my Xbox'. Tom.	`I mean you don't understand if you've got a disability, and you've got an overprotective family, that don't let you play out because they are scared that you are going to get hurt, or that you are going to get picked on, or ye not going to fit in, the only thing you can do is gaming'. Mohsin	'Mm about 6 (hours) from whatever time I get up till when I go to sleep'. Lee	'you don't understand'<br>Contained<br>Protected<br>Placement in the everyday<br>'If I could get out'	When life gives you few choices-video game
